# Genome sequencing of rice subspecies and genetic analysis of recombinant lines reveals regional yield- and quality-associated loci

**DOI:** 10.1186/s12915-018-0572-x

**Published:** 2018-09-18

**Authors:** Xiukun Li, Lian Wu, Jiahong Wang, Jian Sun, Xiuhong Xia, Xin Geng, Xuhong Wang, Zhengjin Xu, Quan Xu

**Affiliations:** 1 Rice Research Institute of Shenyang Agricultural University, Shenyang, 110866 China; 2 Biomarker Technologies Corporation, Beijing, 101300 China

**Keywords:** *Oryza sativa*, De novo assembly, QTL dissection, Yield and quality

## Abstract

**Background:**

Two of the most widely cultivated rice strains are *Oryza sativa indica* and *O. sativa japonica*, and understanding the genetic basis of their agronomic traits is of importance for crop production. These two species are highly distinct in terms of geographical distribution and morphological traits. However, the relationship among genetic background, ecological conditions, and agronomic traits is unclear.

**Results:**

In this study, we performed the de novo assembly of a high-quality genome of SN265, a cultivar that is extensively cultivated as a backbone *japonica* parent in northern China, using single-molecule sequencing. Recombinant inbred lines (RILs) derived from a cross between SN265 and R99 (*indica*) were re-sequenced and cultivated in three distinct ecological conditions. We identify 79 QTLs related to 15 agronomic traits. We found that several genes underwent functional alterations when the ecological conditions were changed, and some alleles exhibited contracted responses to different genetic backgrounds. We validated the involvement of one candidate gene, *DEP1*, in determining panicle length, using CRISPR/Cas9 gene editing.

**Conclusions:**

This study provides information on the suitable environmental conditions, and genetic background, for functional genes in rice breeding. Moreover, the public availability of the reference genome of northern *japonica* SN265 provides a valuable resource for plant biologists and the genetic improvement of crops.

**Electronic supplementary material:**

The online version of this article (10.1186/s12915-018-0572-x) contains supplementary material, which is available to authorized users.

## Background

Rice is one of the most important staple crops in the world and provides more than 20% of the calorie intake for more than half of the world’s population. Given continuing population growth and increasing competition for arable land between food and energy crops, food security is becoming an ever more serious global problem. Two major types of *Oryza sativa japonica* and *O. sativa indica* subspecies have historically been recognized. Varied degrees of geographical distribution and morphology characters exist between the two subspecies. Elucidation of the relationship among the functional genomic of *indica* and *japonica*, ecological conditions, and agronomic traits may significantly contribute to the improvement of rice production. China established a nationwide mega project entitled “Breeding and cultivation system of super rice in China” in 1996. After nearly a decade of cultivation, super rice accounts for more than 60% of the total area under rice cultivation and has contributed an estimated two billion dollars to the Chinese national economy [1, 2]. Shennong 265 (SN265), the first released commercial super rice variety, showed not only erect panicles but also strong root activity and high yield in a range of growing environments. SN265 leads the breeding direction as the backbone parent in northern China. Rice genetics and functional genomics have been rapidly advancing, particularly over the last decade, since the first determination of the Nipponbare genome sequence [3]. To improve understanding of the genetic mechanism of hybrid super rice, the genomes of two elite *indica* rice varieties, namely, Zhenshan 97 and Minghui 63, have been assembled [4]. Recently, a near-complete *indica* rice genome of R498 was published, which enriched the implications for plant biology and crop genetic improvement in *indica* [5]. As the *japonica* varieties in northern China have varying degrees of *indica* pedigree introgression [2], the establishment of a reference for *japonica* in northern China is imperative. Thus, the de novo assembly of the SN265 genome will serve as a reference for the discovery of genes and structural variations that contribute to the increase in rice production in super rice varieties in northern China.

Here, we constructed a high-density linkage map by re-sequencing the recombinant inbred lines (RILs) derived from a cross between the *japonica* variety SN265 and *indica* variety R99. We de novo assembled the two parental genomes of SN265 and R99 based on single-molecule real-time sequencing (SMRT) and high-throughput next-generation sequencing (NGS). The RILs were planted in three areas with distinct ecological conditions, and 15 important agronomic traits were investigated. The re-sequencing and assembly of the parental genomes facilitated QTL analysis and candidate gene identification. The influence of genetic background and ecological condition to gene function was investigated in this study.

## Results

### Population sequencing and linkage map construction

In order to construct the linage map, the RILs derived from the cross between SN265 and R99, along with the parents, were sequenced on an Illumina HiSeq2500 platform. Through the high-throughput sequencing, we obtained a total of 434.37 Gb of clean data, with approximately 6.25-fold depth for each RILs. For parent lines, 30.0-fold depth and 32.0-fold depth data were generated for R99 and SN265, respectively. We aligned these data to the Os-Nipponbare-Reference-IRGSP-1.0 (http://rapdb.dna.affrc.go.jp/download/irgsp1.html) using SOAP2 [6, 7]. Totally 1,708,775 single nucleotide polymorphisms (SNPs) between SN265 and R99 were identified using SOAPsnp [8]. To avoid ambiguity in the analysis, we removed the SNPs that has low genotyping scores or located in highly repetitive regions. As the low-coverage sequencing caused the missing genotype for RILs, the k-nearest neighbor algorithm was used to impute the missing genotypes of each RILs [9]. Subsequently, a recombinant bin map was constructed by 1,456,445 high-quality SNPs. The map contained 3569 recombinant blocks, with the average length of 58.17 kb (Additional file 1: Figure S1 and Additional file 2: Figure S2).

### Assembly of the parental genome

**Fig. 1:**
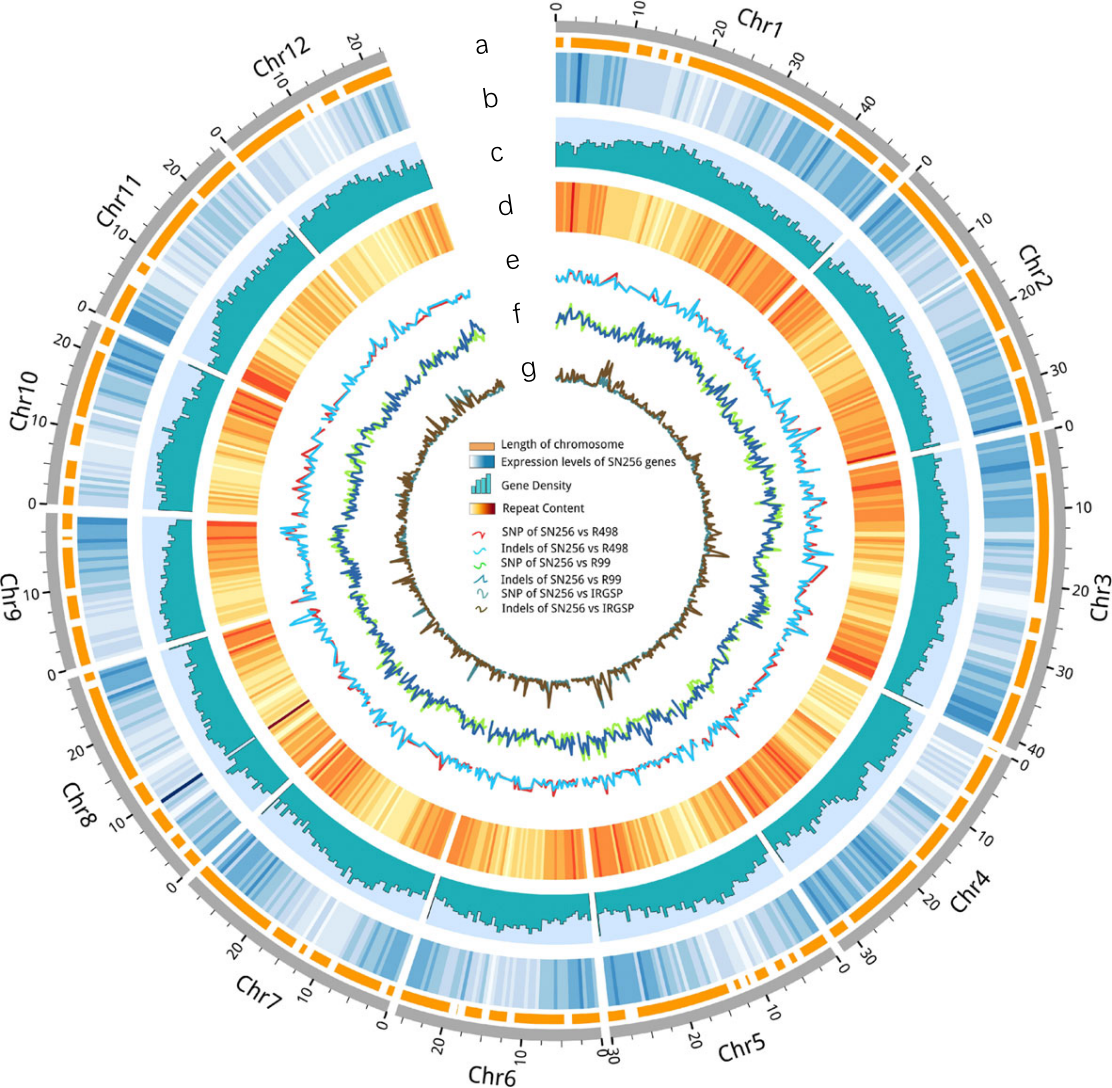
Overview of the SN265 reference genome. Tracks from inner to outer circles indicate the following: a contigs and gaps. b expression level genes, c gene density, d repeat sequence content, e SNPs and indels between SN265 and the *indica* (R498) reference genome, f SNPs and indels between SN265 and the *indica* (R99) reference genome, g SNPs and indels between SN265 and the *japonica* (Nipponbare) reference genome

To fill the gaps in the high-quality genome of super rice of northern China, de novo genome assembly of the parent line, SN265, was performed using RIL populations, real-time sequence (SMRT), high-throughput NGS, and RNA-seq. DNA libraries for SMRT sequencing were constructed as described elsewhere [10]. We generated five SMRT cells using P5-C3 SMRT cell chemistry. A total of 24.94 Gb (60-fold) clean data of subread bases with a mean read length of 9.6 kb were generated after filtering the low-quality and short reads. The high-throughput NGS was performed to polish the assembly. DNA libraries for NGS were constructed with size of 270 bp, a total 22.05 Gb clean data (59.66-fold) had quality scores higher than Q20. A 364.45-Mb SN265 genome with a contig N50 value of 6.96 Mb was assembled (Fig. 1). Only 159 erroneous bases, accounting for 0.0000419% of the contig length, were found. A total of 166.68 Mb of repeat sequence was predicted based on a combination of the primary database constructed in this study and Repbase (Additional file 3: Table S1). In addition, a total of 37,609 genes were obtained by de novo prediction, homologous prediction, and RNA-seq analysis after removing repeat sequences. Finally, 95.17% of the genes were functionally annotated with the NR, KOG, GO, KEGG, and TrEMBL databases (Additional file 4: Table S2 and Additional file 5: Table S3). The predicted motifs, non-coding RNAs, and pseudogenes are shown in Additional file 6: Table S4. We further compared the SN265 genome to the current Os-Nipponbare-Reference-IRGSP-1.0 (http://rapdb.dna.affrc.go.jp/download/irgsp1.html) [7], and SN265 was 18.3 Mb shorter, but had fewer gaps than the Nipponbare genome. SN265 has longer chromosomes than Nipponbare (i.e., Chr.1, Chr.3, Chr.5, and Chr.8) (Table 1). As the reference genome of typical *indica* rice varieties such as R498, Zhenshan 97, and Minghui 63 was recently released [4, 5], we only conducted a lower-fold SMRT to assemble the R99 genome. A total of 13 Gb (30-fold) of clean data with a mean read length of 8.85 kb were generated. We assembled a 389.6-Mb genome with a contig N50 value of 3.05 Mb. Approximately 663 erroneous bases, accounting for 0.000196% of the contig length, were detected. A total of 166.68 Mb of repeat sequences were predicted, and 36,089 genes were obtained (Additional file 3: Table S1). Finally, 99.92% of the genes were functionally annotated with the NR, KOG, GO, KEGG, and TrEMBL databases (Additional file 4: Tables S2 and Additional file 5: Table S3).

**Table 1:** Comparison of basic sequence statistic of SN265 and Os-Nipponbare-Reference-IRGSP-1.0

Chr.	SN265 length	Nip length	SN265 gaps	Nipponbare gaps
Chr1	48,728,733	45,038,628	7	8
Chr2	35,824,122	36,792,250	2	5
Chr3	41,114,166	37,312,367	4	8
Chr4	32,130,185	35,923,694	5	9
Chr5	30,181,130	30,073,434	9	5
Chr6	26,064,998	32,124,787	5	4
Chr7	29,637,245	30,324,621	5	3
Chr8	28,695,623	28,530,022	6	3
Chr9	19,884,354	23,895,720	4	7
Chr10	23,682,709	23,880,549	6	9
Chr11	25,682,812	31,198,810	6	6
Chr12	22,821,698	27,676,856	4	5
Total	364,447,775	382,771,738	63	72

### *Indica* pedigree percentage affects yield and quality traits

**Fig. 2:**
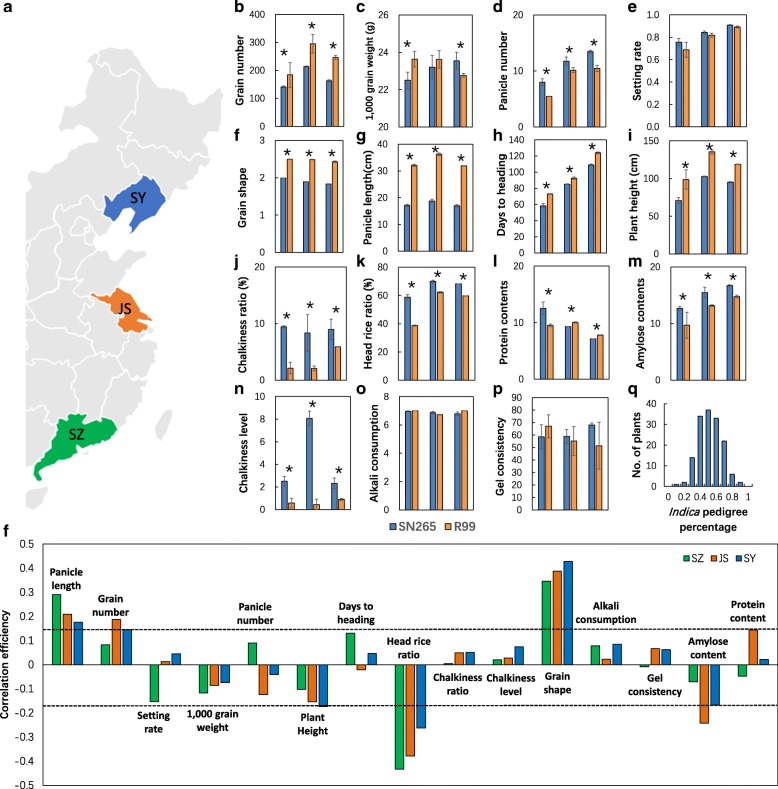
The yield components and quality traits of parental lines in three areas: **a** the location of three cultivated areas, **b** grain number, **c** 1000 grain weight (**g**), **d** panicle number, **e** setting rate, **f** grain shape, **g** panicle length (cm), **h** days to heading, **i** plant height (cm), **j** chalkiness rice ratio (%), **k** head rice ratio, **l** protein content, **m** amylose content, **n** chalkiness level, **o** alkali consumption, **p** gel consistency, **q** *indica* pedigree percentage of RILs, and **r** the correlation coefficient of *indica* pedigree percentage to yield components and quality. The data are the mean ± s.d. (*n* = 20 plants), asterisks, and dotted line, significant at the 5% level

To elucidate the relationship between *indica*/*japonica* genetic background and agronomic traits, we determined the *indica* pedigree percentage of each RIL using sequence data. The *indica* pedigree percentage was defined as the ratio of the number of *indica*-type SNPs to all of the subspecies-specific SNPs for each RILs. The subspecies-specific SNPs were those of the same type in all *japonica*, but not in *indica*, which is based on the divergence of the 517 rice landraces [9]. In total, 100,529 subspecies-specific SNPs were selected. We matched the 1,794,441 SNPs between SN265 and R99 to 100,529 subspecies-specific SNPs, and 61,920 SNPs were merged. The 61,920 SNPs were then used for *indica* pedigree percentage analysis. The results showed that the *indica* pedigree percentage of the RILs followed a normal distribution (Fig. 2). We conducted a correlation analysis of *indica* pedigree percentage to the yield and quality traits. The results showed that the *indica* pedigree percentage showed a significant positive correlation to panicle length and grain shape (the ratio of grain length to grain width) and a negative correlation to head rice ratio in all three of the areas. With increasing latitude, the correlation efficiency between *indica* pedigree percentage and grain shape became larger, and the correlation efficiency of *indica* pedigree percentage to panicle length and head rice ratio became smaller. In JS, the *indica* pedigree percentage also has a significant positive correlation to grain number and a significant negative correlation to amylose content (Fig. 2). As the grain shape always had a significant negative correlation to head rice ratio, we concluded that the *indica* pedigree percentage mainly affects panicle length and grain shape.

### QTL detection and analysis using the RIL population

**Fig. 3:**
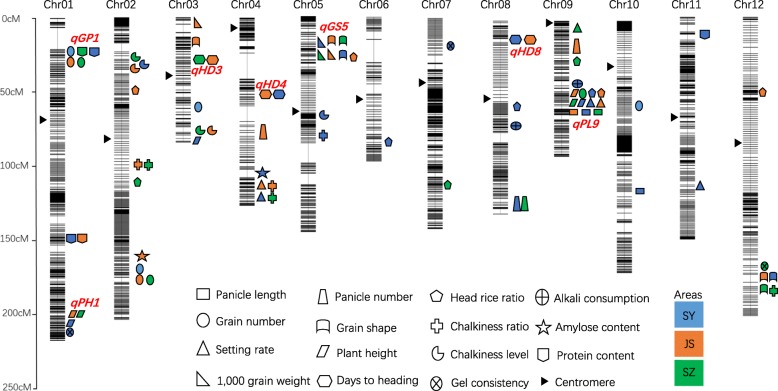
The positions of 79 QTLs for yield components and quality traits located on each chromosome. Different colors represent the QTLs detected in various areas

To elucidate the genetic mechanism underlying yield and quality, we primarily focused on 15 traits (panicle length, panicle number, heading rice ratio, alkali consumption, grain number, grain shape, chalkiness ratio, amylose content, setting rate, plant height, chalkiness level, protein content, 1,000-grain weight, days to heading, and gel consistency) known to be important for rice yield and quality. Both SN265 and R99 showed significant differences in all of the traits except for setting rate, alkali consumption, and gel consistency (Fig. 2). Based on sequence variations in SN265 and R99 and the variant sequences among the RILs, 1,456,445 SNPs loci were used for linkage analysis. The loci that co-segregated with one another were anchored to the same blocks and designated as “bins.” A total of 3569 bins were used to construct a molecular linkage map using Highmaps software. The phenotype datasets for the RILs collected in the three areas were used for QTL analysis. A total 79 QTLs for all of the traits were mapped independently on rice chromosomes 1 to 12 (Fig. 3 and Additional file 7: Table S5). Among the QTLs, several QTL clusters are highlighted: one locus for grain number per panicle, setting rate, and alkali consumption on the short arm of chromosome 1; one locus for plant height and gel consistency on the long arm of chromosome 1; one locus for grain shape, 1000-grain weight, chalkiness ratio, and alkali consumption on the short arm of chromosome 5; and one locus for plant height, panicle length, grain number per panicle head, and rice ratio on the long arm of chromosome 9. These results suggest that these QTL clusters may be controlled either by one gene with pleiotropy or by a group of closely linked genes. We also found that some loci can be detected in all three of the areas, whereas others can only be detected in only one or two areas.

### Fine-mapping of QTLs for QTL clustering

**Fig. 4:**
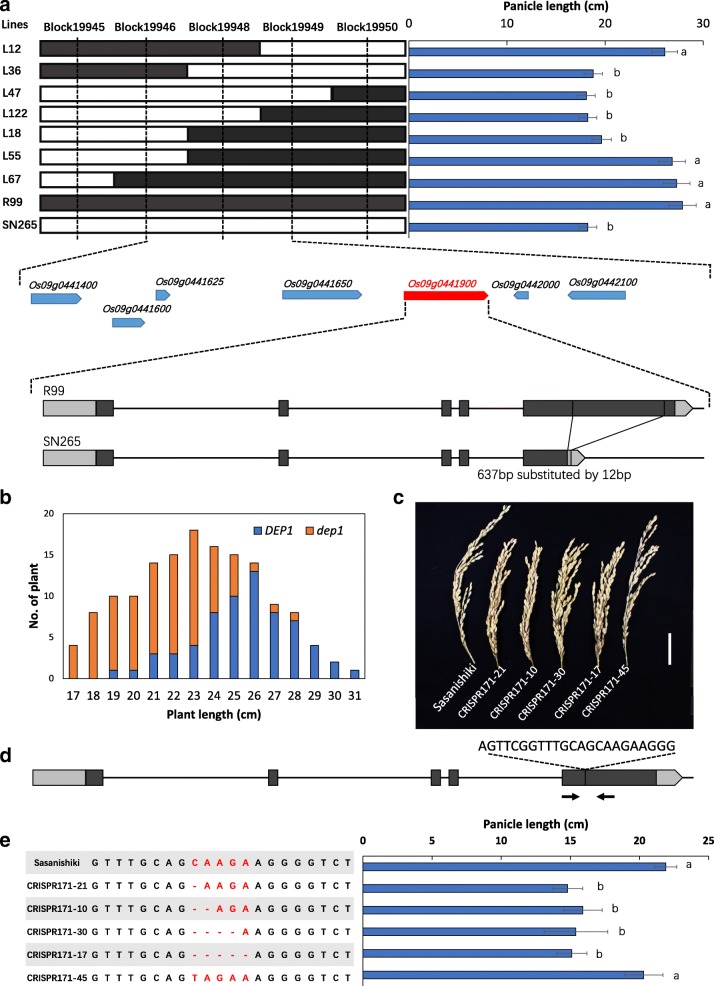
Fine-mapping and CRISPR/Cas9 gene editing of *qPL9/DEP1*. **a** Fine-mapping and sequence comparison of *qPL9*. **b** Co-separation of *DEP1* in RILs, the upper and lower letters indicate the R99-type and SN265-type alleles, respectively. **c** The panicle length of CRISPR/Cas9 gene editing plants and Sasanishiki (WT). **d** Schematic diagram of the genomic region of *qPL9* and the sgRNA target site. **e** Sequence alignment of the sgRNA target region showing altered bases in different mutant lines and the panicle length of mutant lines. The data are the mean ± s.d. (*n* = 20 plants), and the scale bar is 4 cm

**Fig. 5:**
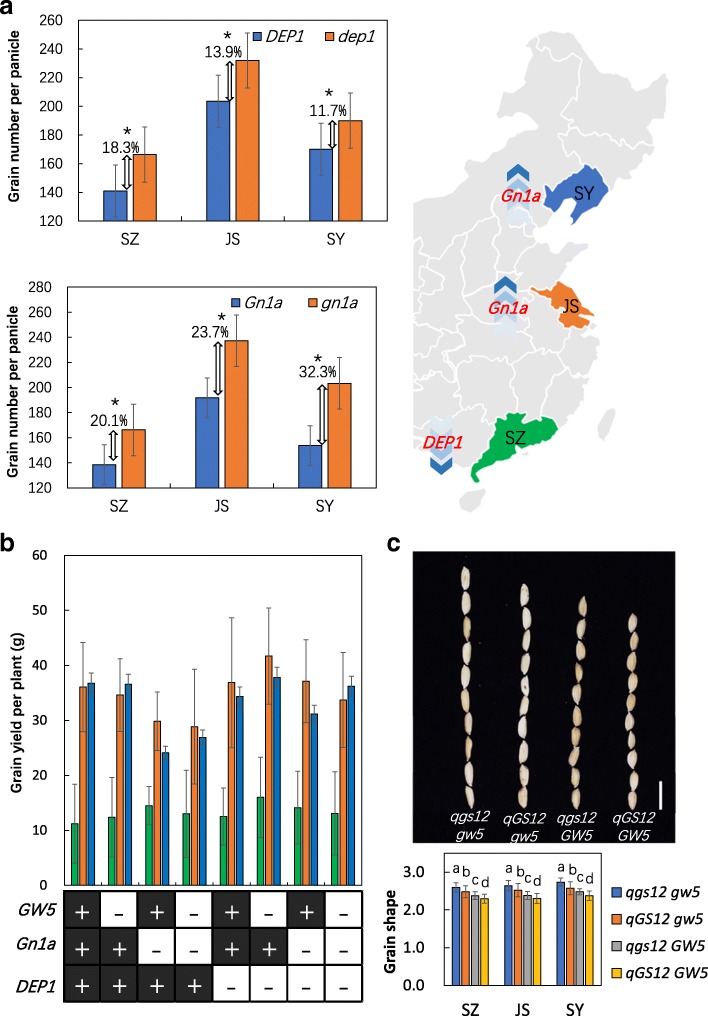
The effects of *DEP1* and *Gn1a* in RILs at low-, middle-, and high-latitude areas. **a** The effect of *DEP1* and *Gn1a* to grain number per panicle in different areas. Asterisks are significant at the 5% level. **b** The effect of the combination among *GW5*, *Gn1a*, and *DEP1* on grain yield per plant in the three areas. **c** The effect of the *qGS12* and *GW5* on grain shape in three areas. Plus sign and lowercase letters indicate the *indica*-type (R99) allele; the minus sign and uppercase letters indicate the *japonica*-type (SN265) allele. The data are the mean ± s.d. (*n* = 20 plants), and the scale bar is 1 cm

To identify candidate genes for the QTL cluster, we conducted fine-mapping using the genome assembly of the two parental lines. We first focused on the *qPL9* cluster on chromosome 9, and the panicle length data in SY was used for fine-mapping. The candidate gene was mapped to a 43-kb interval in block 19948 (Fig. 4). Seven annotated genes were present in this bin. We compared the sequence of seven annotated genes between SN265 and R99 using the assembled genome, revealed a replacement of a 637-bp stretch in the middle region of exon 5 by a 12-bp sequence in SN265 at *DENSE AND ERECT PANICLE 1* (*DEP1*) locus (Fig. 4). *DEP1* has been previously shown to be a pleiotropic major QTL responsible for grain number and panicle architecture [11]. Then, we conducted a co-segregation analysis of *DEP1*, which showed that plants harboring the SN265-type allele of *dep1* develop shorter panicles (Fig. 4). To confirm that *DEP1* is a candidate gene for *qPL9*, we conducted a mutagenesis assay involving *DEP1* in the *japonica* cultivar Sasanishiki using the CRISPR/Cas9 technology. After introducing the construct into rice embryogenic calli by *Agrobacterium*-mediated transformation, we obtained at least 30 independent regenerated transgenic lines. The mutant lines were grown in the field, and the *T*_2_ mutants were sequenced. We detected five homozygous mutants, and the sequencing results are presented in Fig. 4. Among the five mutant lines, four lines harbored a deletion within exon 5, which is predicted to result in a frame shift. One line carried a sequence substitution that was predicted to lead to an amino acid change. We investigated the panicle length in these mutant lines at the mature stage. The results showed that the panicle length in the four lines that harbored a frame shift mutation had significantly decreased compared to Sasanishiki, whereas the substitution line showed a similar panicle length to that in Sasanishiki (Fig. 4). Next, we identified the candidate gene of *qGP1* cluster on the short arm of chromosome 1. The number of grains in SY was used to conduct fine-mapping. The candidate gene was fixed in *Gn1a* [12], and sequence analysis showed that one G/C SNP and a 6-bp deletion occurred in the first exon of R99 compared to the sequence of SN265 (Additional file 8: Figure S3). Then, we identified *SD1* as the candidate gene that corresponds to plant height on the long arm of chromosome 1 [13], and the sequence of first exon significantly differed between R99 and SN265. We identified *GW5* as the candidate gene for grain shape, 1000 grain weight, chalkiness ratio, and alkali consumption and localized this to the short arm of chromosome 5 [14, 15]. A 1,212-bp deletion on the 5.7-kb upstream region of *GW5* was detected in SN265 relative to that in R99 (Additional file 9: Figure S4). We also detected in *qGS12* on chromosome 12, which explains 15.44% of the observed variation. QTL analysis mapped *qGS12* to Block7839, which includes a 23.74-kb region. There were three putative genes within this region, and sequence analysis only detected a single SNP in the third exon of *Os12g0610600* (Additional file 10: Figure S5). *Os12g0610600* was reported as a NAC transcription factor that negatively regulated drought tolerance in rice [16]. The NAC transcription factor family is involved in a number of biological processes in rice, such as drought and salt tolerance, resistance to bacterial leaf blight, heading time, and ABA biosynthesis [17]. The grain shape exhibited significant differences among the combinations between *GW5* and *qGS12* (Fig. 5). Our results show that NAC transcription factors may participate in the regulation of grain shape. However, additional complementary testing as well as supporting genetic evidence is warranted. Moreover, we found that *DTH8*, *SDG708*, and *phytochrome B (PHYB)* were the candidate genes for the heading date QTL on chromosomes 8, 4, and 3, respectively [18–20]. There were two SNPs at the first exon region between SN265 and R99 in the *PHYB* locus, a T/G SNP and 19-bp deletion on the *DTH8* locus in R99 and multiple changes leading to a truncated gene structure at the 3′ terminal of *SDG708* in R99. The sequence differences in these candidate genes between SN265 and R99 are shown in Additional file 9: Figure S4.

### The influence of ecological conditions and genetic background to gene function

As the RILs were derived from the cross between *indica* and *japonica* and we cultivated the RILs into three areas with distinct ecological conditions, we were able to elucidate the influence of ecological conditions and genetic background to gene function. First, we found that *Gn1a* largely contributed to grain number per panicle in SY and JS, but not in SZ. However, QTL analysis only detected *DEP1* in SZ as a grain number per panicle QTL, but not in SY and JS. We further compared the plant carrying *DEP1/dep1* and *Gn1a/gn1a* in the three areas, which showed that with increasing latitude, the effect of *dep1* on increasing grain number became weaker, whereas the effect of *gn1a* on increasing grain number became stronger (Fig. 5). Similarly, QTL analysis can detect *DTH8*, *SDG708*, and *PHYB* in JS, but only *PHYB* in SZ, and only *SDG708* and *DTH8* in SY. We selected different gene combinations of *DTH8*, *SDG708*, and *PHYB* in the RILs and compared the heading date of these lines in the three areas. The results confirmed that *DTH8* and *SDG708* barely affect heading date in SZ, and the *PHYB* imparted weaker effects on SY (Additional file 11: Figure S6). In summary, the function of *Gn1a*, *SDG708*, *SD1*, and *GW5* was enlarged with increasing latitude. On the other hand, a disruption in the function of *PHYB* and *DEP1* was observed with increasing latitude. Interestingly, *DTH8* showed the strongest function in middle latitudes and became weaker with increasing or decreasing latitude.

Then, we analyzed the influence of *indica* pedigree percentage to gene function. The RILs were divided into three groups based on the *indica* pedigree percentage: *japonica* group (*indica* pedigree percentage 0~0.4), inter-group (*indica* pedigree percentage 0.4~0.6), and *indica* group (*indica* pedigree percentage 0.6~1). We found that gene function also differs with genetic background. Interestingly, the *japonica*-type allele of *SD1* induces a decrease in plant height with increasing *indica* pedigree percentage, whereas the *indica-*type *sd1* allele increases plant height with higher *indica* pedigree percentage (Additional file 12: Figure S7). Moreover, the *japonica*-type allele of *PHYB* delays the heading date with increasing *indica* pedigree in SZ, but accelerates heading date in JS and SY. Additionally, several gene functions in the inter-type group showed worst agronomic traits among three groups. For example, the inter-group exhibited the lowest grain number per panicle under a *DEP1* and *Gn1a* genetic backgrounds (Additional file 13: Table S6). This may explain why the rare commercial varieties have the inter-type genetic background.

## Discussion

Rice is a short-day plant model that flowers more rapidly in short-day conditions exhibit delayed flowering under long-day conditions, thereby indicating the existence of critical day length responses [20]. In the wild-type plants, although *Hd3a* mRNA is highly expressed at day lengths ≤ 13 h, its expression markedly decreased to about one-tenth of the expression, at a day length of 13.5 h and became undetectable at day lengths of ≥ 14 h [21]. Our previous study has monitored day length and the temperature during the whole growth season in all three areas in 2016 [22]. We observed RIL growth under long-day conditions for the whole growing season in SY and JS, and the day length even exceeded 15 h in SY at July. However, the entire growth period of the RILs involved short-day conditions in SZ (Additional file 14: Figure S8). Thus, we assumed that the photoperiod-sensitive genes play supporting roles in SZ as it maintains optimal day length possibly for the entire growth season, and other factors, such as temperature, play a lead role in establishing heading date. Then, we analyzed the relationship between temperature and heading date in SZ using the method described in our previous study [22]. We found that temperature has a strong correlation with heading date, and this correlation exhibited a positive/negative change by a 10-day rhythm (Additional file 14: Figure S8). Ambient temperature regulates various aspects of plant growth and development, but the actual indicators in rice remain elusive. In *Arabidopsis*, in addition to its photoreceptor function, *PHYB* acts as a temperature sensor [23]. As *PHYB* was the only detected QTL corresponding to heading date in SZ, we assumed that *PHYB* may also be involved in the temperature responses in rice.

Grain weight is a major determinant of crop grain yield and is controlled by naturally occurring quantitative traits loci. Grain shape largely differed between *indica* and *japonica*. *GW5* was detected in most *japonica* cultivars during rice domestication, and a 1212-bp deletion was associated with the increased grain width in *japonica* cultivars [24]. The present study confirmed that the 1212-bp deletion 5.7-kb upstream of *GW5* was the major factor affecting grain width, which explains 38.26% of the observed variation. The SN265-type truncated *dep1* allele is widely distributed among *japonica* varieties in Northeast China and the Yangtze River area [25]. Moreover, the function of the *indica*-type *gn1a* is enhanced with increasing latitude. These results suggest that the introduction of the *japonica* elite allele into the *indica* genetic background or cultivated zone may improve the agronomic traits of *indica* and vice versa. The erect panicle architecture caused by the *dep1* allele significantly increases grain yield; however, the quality traits of these varieties are only considered to be mediocre. As grain width is always significantly positively correlated to chalkiness level, the combination of the *indica*-type *gw5* and *dep1* alleles can simultaneously improve grain yield and morphological traits. Moreover, genotypic analysis of QTLs demonstrated that the haplotype status in RIL lines is responsible for the corresponding traits, whereas the combination of favorable QTLs contributes to relatively high yield per plant. The combination of the SN265-type allele of *DEP1*, *GW5*, and *indica*-type of *Gn1a* is associated with the highest grain yield per plant in all three of the areas (Fig. 5).

With the application of high-throughput sequencing technology, numerous rice accessions have been re-sequenced and phenotyped in the past few years, allowing the exploration of genomic diversity, particularly in terms of identifying loci that are responsive to domestication, as well as in elucidating the molecular mechanism underlying important agronomic traits [9, 26–29]. The de novo assembly of the rice genome provides us with more information to comprehensively capture the genomic diversity in this species [30]. In this study, we performed de novo assembly of a 364.45-Mb SN265 genome as a reference for super rice in northern China using an RIL population, real-time sequencing (SMRT), and high-throughput NGS.

## Conclusions

Our study identified 79 QTLs that are related to the 15 agronomic traits in three areas with distinct ecological condition and found that several genes underwent functional alterations when the ecological conditions and genetic background were altered. We de novo assembly a super rice variety SN265, and the availability of high-quality reference genomes for the *japonica* subspecies not only facilitates the identification of genes corresponding to agronomic traits but also provides a range of implications for plant biology and crop genetic improvement.

## Methods

### Plant materials and quality measurements

We conducted a cross between “Shennong265” (*Oryza sativa japonica*) and “R99” (*O. sativa indica*) and used the single-seed descendant method to generate RILs with at least 10 generation inbred. A total of 151 RILs were constructed and were used in this study. Field experiments were conducted in three typical rice cultivated areas: the Agricultural Genomics Institute at Shenzhen (SZ; N22°, E114°), the sub-base of China National Hybrid Rice R&D Center in Jiangsu Province (JS; N32°, E120°), and the Rice Research Institute of Shenyang Agricultural University (SY; N41°, E123°) for two growing seasons during 2015–2016. The cultivation method and field management were described in our previous report [22]. We harvested the field examination plants at 45 days after heading for each line in each of the three areas. A total of 20 plants from the middle rows were harvested for each line. The quality measurement was conducted as described in our previous study [22]. We only used the 2016 data in the present study, as the 2 years of data showed similar trends and are highly correlated. All samples were analyzed with two biological replicates.

### DNA extraction and re-sequencing

We sampled the young leaves for each lines 2 weeks after transplanting. To obtain the high-quality DNA, the cetyltrimethylammonium bromide (CTAB) method was used to extract genomic DNA. The sequencing libraries were constructed on the Illumina HiSeq2500 following the manufacturer’s instructions. We aligned the sequencing data to the *japonica* reference genome (Nipponbare, http://rapdb.dna.affrc.go.jp/download/irgsp1.html/) using SOAP2 [6]. To construct the genetic map, we combined the co-segregating markers (SNP and/or InDel) into bins using HighMap software [31]. The constructed map contained 3569 bins, and there were average 247 bins on each chromosome. The map contained 1965.33 cM genetic distance. There were 12 linkage groups in the linkage map, which correspond to the 12 rice chromosomes. The full collinearity between the genetic map and the rice genome was observed, as the minimum value of spearman coefficient for chromosome was 0.9725 (Chr. 6).

### Single-molecule real-time sequencing (SMRT) and high-throughput NGS

The genomic DNA of each line was extracted from fresh leaves using DNeasy Plant Mini kits (Qiagen, Germany) according to manufacturer’s instructions. DNA libraries for SMRT sequencing were performed as described elsewhere [10]. The single-molecule sequencing (SMS) data are assembled following a hierarchical approach: (1) select a subset of longer reads as seed data and correct through canu/falcon [32], (2) use the error-corrected reads for a draft assembly by different assemblers, and (3) polish the draft assembly using Quiver/Arrow and Pilon. In the correction approach, Canu first selects longer seed reads with the settings “genomeSize = 1000000000” and “corOutCoverage = 80,” then detects raw reads overlapping through high-sensitive overlapper MHAP (mhap-2.1.2, option “corMhapSensitivity = normal”), then finally performs an error correction through falcon_sense method (option “correctedErrorRate = 0.025”). In the next approach, with the default parameters, error-corrected reads are trimmed unsupported bases and hairpin adaptors to get the longest supported range. In the last approach, Canu generates the draft assembly by the longest 80 coverage trimmed reads. The draft assembly is polished to obtain the final assembly. Two rounds of polishing are conducted. The first round polishing adopts arrow algorithm by SMS data with the 40 threads, and the second polishing adopts pilon algorithm (v1.22, available at https://github.com/broadinstitute/pilon) using illumina data with the parameters “--mindepth 10 --changes --threads 4 --fix bases.”

### Gene annotation

The RNAs of SN265 and R99 were isolated from the fresh leaves using a TaKaRa MiniBEST Universal RNA Extraction Kit according to manufacturer’s protocol. The sequencing was performed using the Illumina HiSeq 2500 platform according to manufacturer’s instructions. We obtained 8 Gb of RNA-seq data. The MITE-Hunter, LTR_FINDER v1.05, RepeatScout v1.0.5, and PILER-DF v2.4 were used to construct a primary repeat sequence database using structural prediction and ab initio prediction theory [33–36]. We classified the primary database based on PASTE Classifier and then combined with the Repbase database to form the final repeat sequence database for the final prediction through Repeat Masker v4.0.6 [37–39]. In protein-coding gene prediction, the repeat elements were masked and excluded from the genome assembly. Gene annotation was performed by three prediction steps: (1) ab initio prediction using Augustus v2.4, Genscan, GlimmerHMM v3.0.4, GeneID v1.4, and SNAP (version 2006-07-28); (2) homologous species prediction based on *Oryza sativa*, *Arabidopsis thaliana*, *Setaria italica*, *Sorghum bicolor*, and *Zea mays* using GeMoMa v1.3.1; and (3) unigene prediction based on full-length transcriptome data assembly with no reference genome was conducted through PASA v2.0.2 [40–45]. The three predictions were integrated through EVM v1.1.1, and final modifications were performed by PASA v2.0.2 [46]. Non-coding RNAs (microRNAs, rRNAs, and tRNAs) were identified with different strategies according to their unique structural features. The miRBase, Rfam, and tRNAscan-SE v1.3.1 databases were used to predict microRNA, rRNA, and tRNA, respectively [47, 48]. We predicted pseudogenes through scanning for homologous genes and excluding genuine genes by GenBlastA v1.0.4 [49]. We selected the candidate genes with premature stop codons and frameshift mutations as the final pseudogene predictions by GeneWise v2.4.1 [50]. In order to annotate genes’ function, we blasted the predicted genes to the NR, KOG, GO, TrEMBL, and KEGG databases by BLAST v2.2.31 (-evalue 1e-5) [50–55]. In addition, the motifs were annotated according to the sequence alignments with the HAMAP, Pfam, PRINTS, ProDom, SMART, TIGRFAMs, SUPERFAMILY, PIRSF, CATH-Gene3D, and PANTHER databases by InterProScan software [56].

### Vector construction and plant transformation

To conduct the CRISPR/Cas9 gene editing, we performed the vector construction as described by Li et al. [57]. The targeting sequence including PAM sequence (23 bp) was selected in the 5th exon of *DEP1* gene. We confirmed the specificity of targeting sequence by BLAST searching against the rice genome (http://blast.ncbi.nlm.nih.gov/Blast.cgi) [58]. We performed rice transformation as described elsewhere [59]. We extracted the genomic DNA from transformants, and the genomic DNA were sequenced for mutant identification. The PCR products (200–500 bp) were sequenced and identified using the Degenerate Sequence Decoding method [60].

## Additional files

Additional file 1: **Figure S1.** Graphic representation of the genotypes of 151 RILs that were identified using a sliding window approach along each chromosome. Different colors represent different genotypes: red, R99; blue, SN265; yellow, heterozygous blocks. (PDF 23 kb)

Additional file 2: **Figure S2.** Collinearity between the bin map derived from the RIL population and the reference genome (Nipponbare). The horizontal and the vertical axes represent the genetic position of the 12 linkage groups from the RIL population map and the physical positions of the 12 rice chromosomes, respectively. The scattered points represent the bin markers used in QTL mapping. The data for each chromosome and linage group pair are the Spearman correlation coefficient values, which indicated good collinearity when it approaches 1. (PDF 100 kb)

Additional file 3: **Table S1.** Repeat sequence prediction statistics in SN265 and R99. (XLSX 12 kb)

Additional file 4: **Table S2.** Gene prediction statistics of SN265 and R99. (XLSX 9 kb)

Additional file 5: **Table S3.** Gene annotation statistics of SN265 and R99. (XLSX 9 kb)

Additional file 6: **Table S4.** Predicted non-coding RNAs, pseudogenes, and motifs. (XLSX 9 kb)

Additional file7: **Table S5.** The yield components and quality traits of *japonica*, intermediate type and *indica*-type plants in the three areas. (XLSX 14 kb)

Additional file 8: **Figure S3.** Fine-mapping and sequence comparison of *qGP1*(*Gn1a*): a, the qGP1 was mapped between to Block244; b, the annotated genes inBlock244; and c, the sequence difference of Gn1a between SN265 and R99. (PDF 103 kb)

Additional file 9: **Figure S4.** Sequence comparison of *PHYB*, *SD1*, *DTH8*, *SDG708*, and *GW5* between SN265 and R99. (PDF 47 kb)

Additional file 10: **Figure S5.** Candidate gene prediction of grain shape regulate locus on chromosome 12. (PDF 23 kb)

Additional file 11: **Figure S6.** The effects of *DTH8*, *SDG708*, and *PHYB* in different areas: a, the heading date of the different combination of *DTH8*, *SDG708*, and *PHYB*; b, the major effect heading gene in three areas. The data are the mean ± s.d. (*n* = 20 plants), “+” and “−” indicate the R99-type and S265-type alleles, respectively. (PDF 185 kb)

Additional file 12: **Figure S7.** The influence of ecological conditions and genetic background on gene function. The uppercase and lowercase lettered gene name indicates R99-type and SN265-type alleles, respectively. (PDF 378 kb)

Additional file 13: **Table S6.** The QTL analysis for 15 agronomic traits in the three areas. (XLSX 12 kb)

Additional file 14: **Figure S8.** The heading time of RILs in the three areas: a, the heading data of RILs and the day length in three areas; b, the correlation coefficient between air temperature and heading time. (PDF 109 kb)
