# Supplementary figures and images for: Genome sequencing of rice subspecies and genetic analysis of recombinant lines reveals regional yield- and quality-associated loci
Source: BMC Biol. 2018 Sep 18;16:102. doi: 10.1186/s12915-018-0572-x (PMC6145349; doi:10.1186/s12915-018-0572-x)

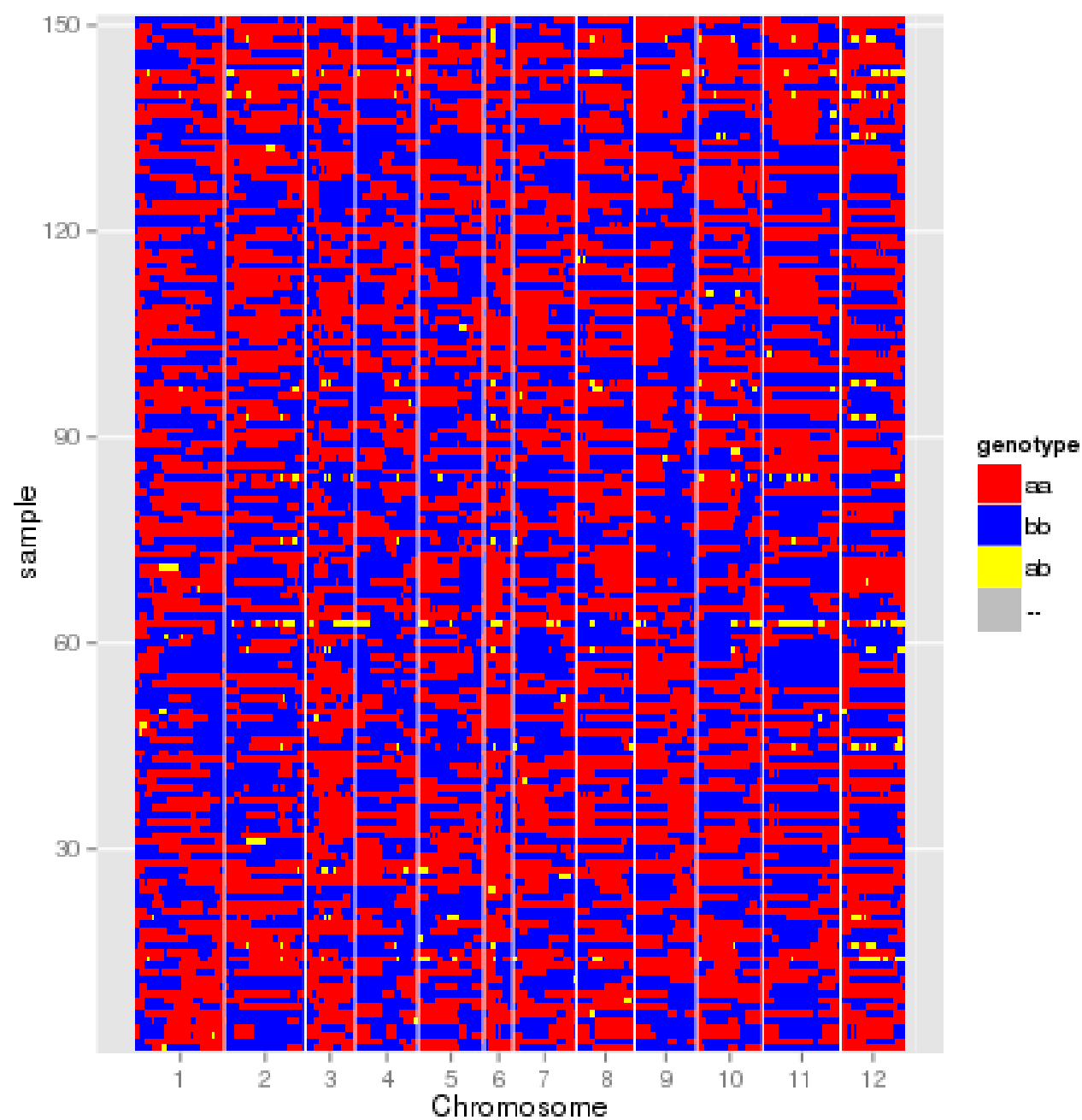

Supplement: Supplementary file 1 — Figure S1. Graphic representation of the genotypes of 151 RILs that were identified using a sliding window approach along each chromosome. Different colors represent different genotypes: red, R99; blue, SN265; yellow, heterozygous blocks. (PDF 23 kb) [file 12915_2018_572_MOESM1_ESM.pdf]

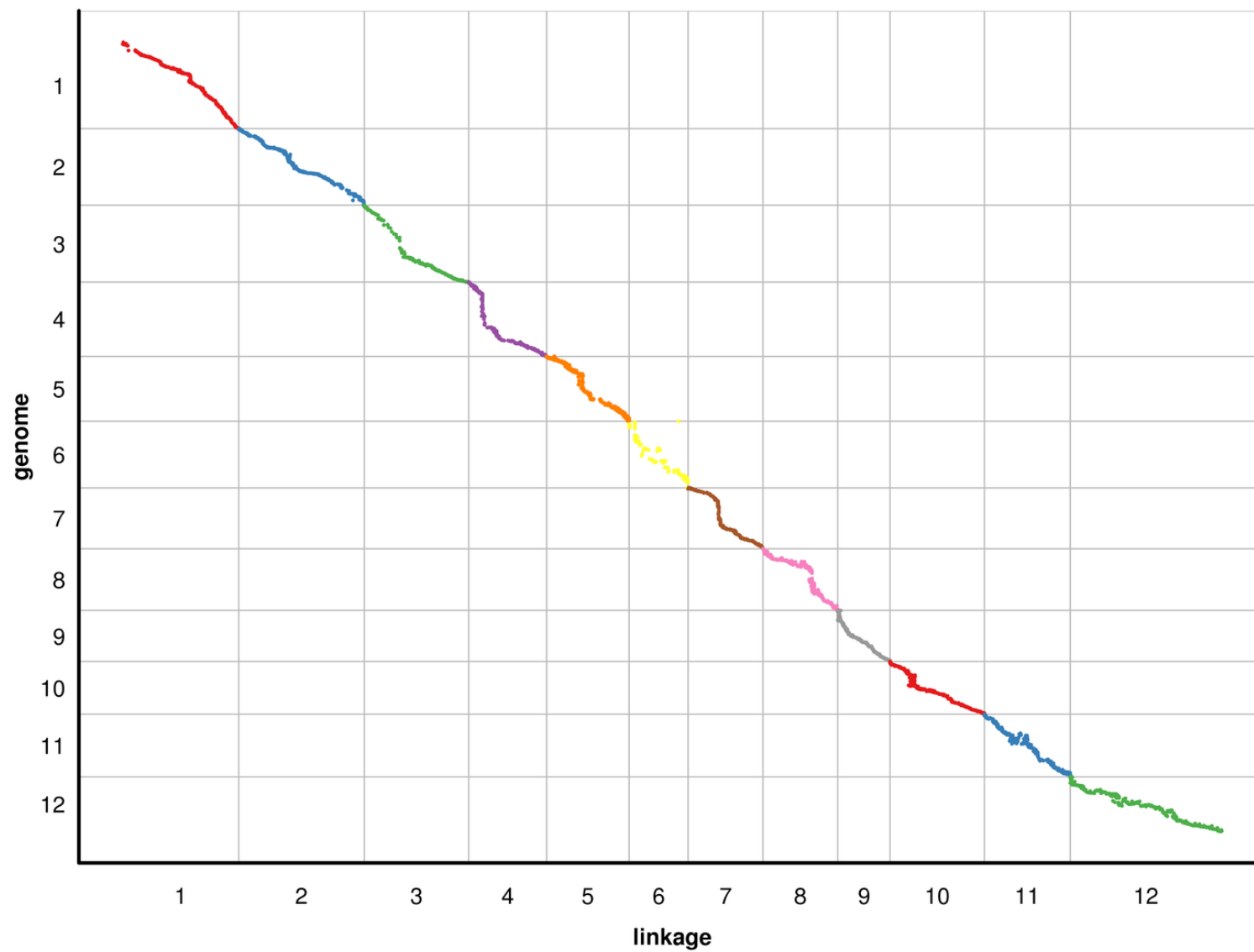

Supplement: Supplementary file 2 — Figure S2. Collinearity between the bin map derived from the RIL population and the reference genome (Nipponbare). The horizontal and the vertical axes represent the genetic position of the 12 linkage groups from the RIL population map and the physical positions of the 12 rice chromosomes, respectively. The scattered points represent the bin markers used in QTL mapping. The data for each chromosome and linage group pair are the Spearman correlation coefficient values, which indicated good collinearity when it approaches 1. (PDF 100 kb) [file 12915_2018_572_MOESM2_ESM.pdf]

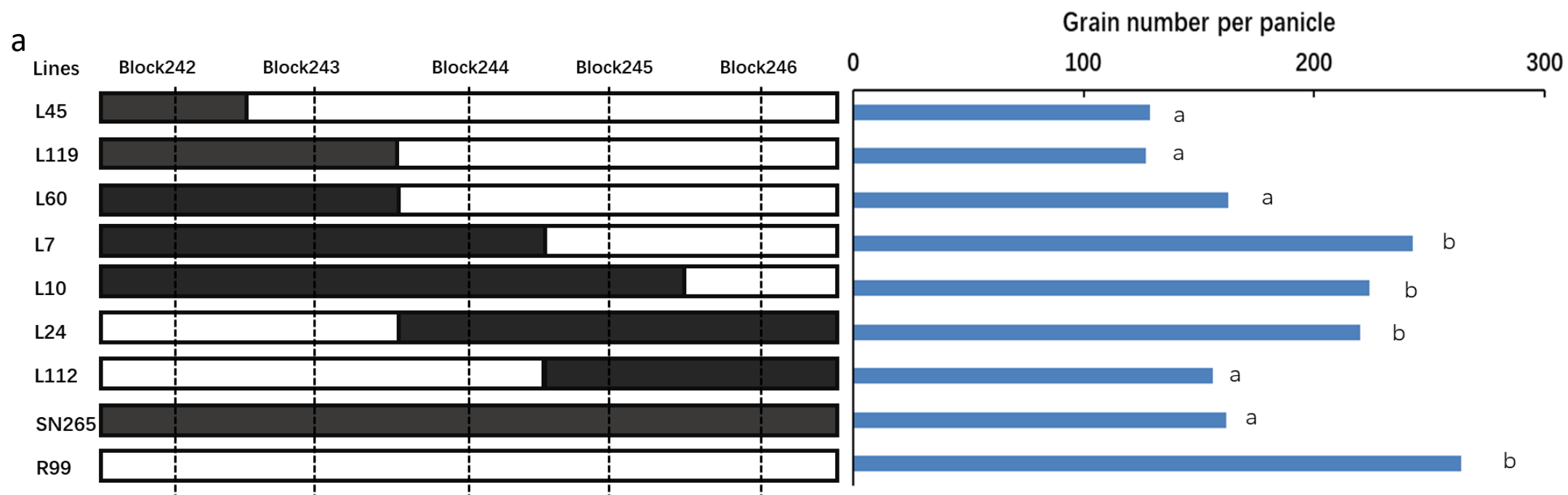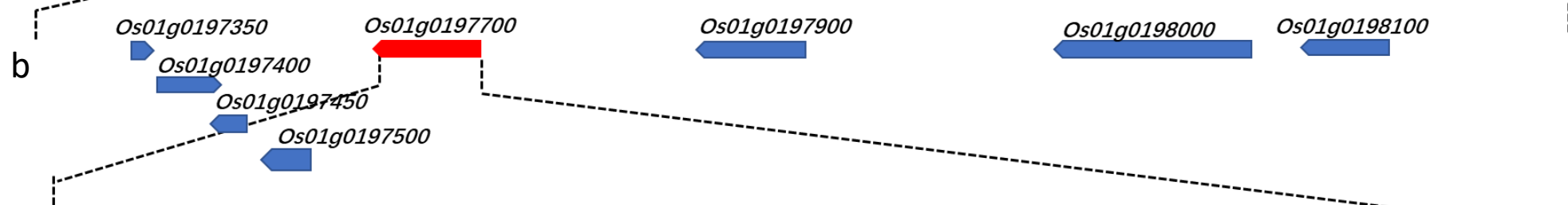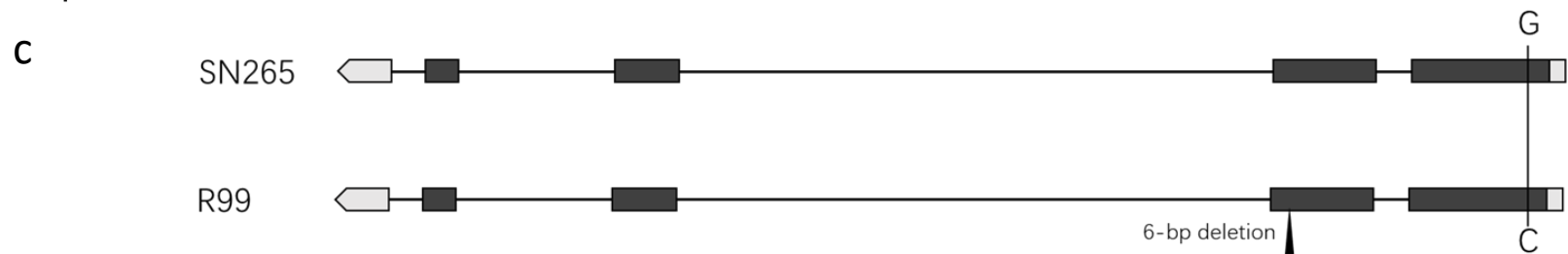

Supplement: Supplementary file 8 — Figure S3. Fine-mapping and sequence comparison of qGP1(Gn1a): a, the qGP1 was mapped between to Block244; b, the annotated genes inBlock244; and c, the sequence difference of Gn1a between SN265 and R99. (PDF 103 kb) [file 12915_2018_572_MOESM8_ESM.pdf]

*PHYB*

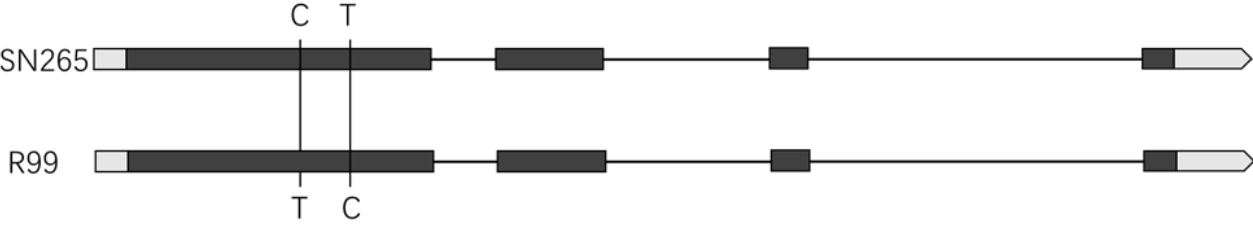

*SD1*

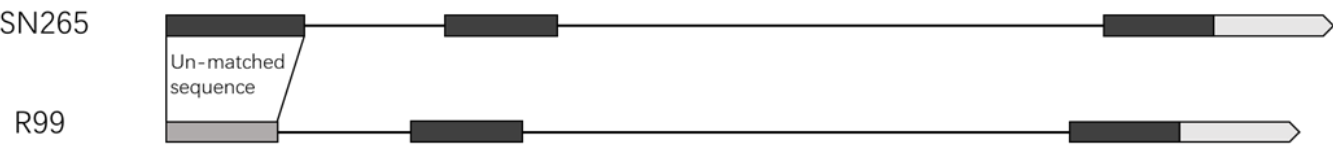

*DTH8*

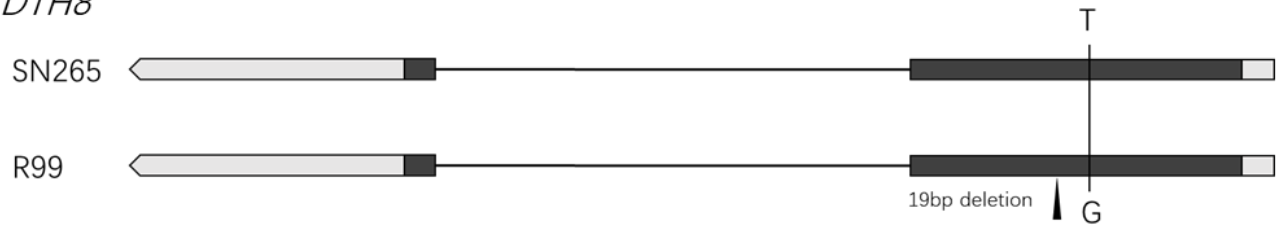

*SDG708*

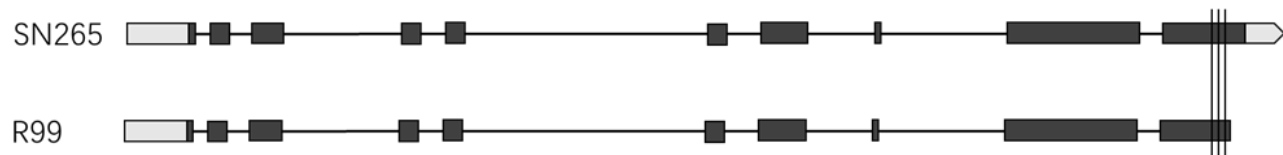

*GW5*

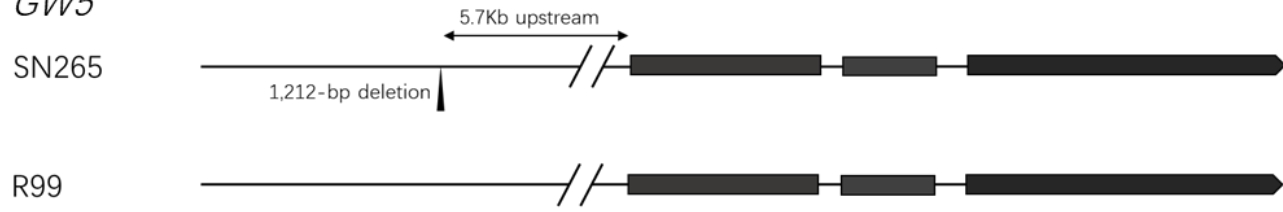

Supplement: Supplementary file 9 — Figure S4. Sequence comparison of PHYB, SD1, DTH8, SDG708, and GW5 between SN265 and R99. (PDF 47 kb) [file 12915_2018_572_MOESM9_ESM.pdf]

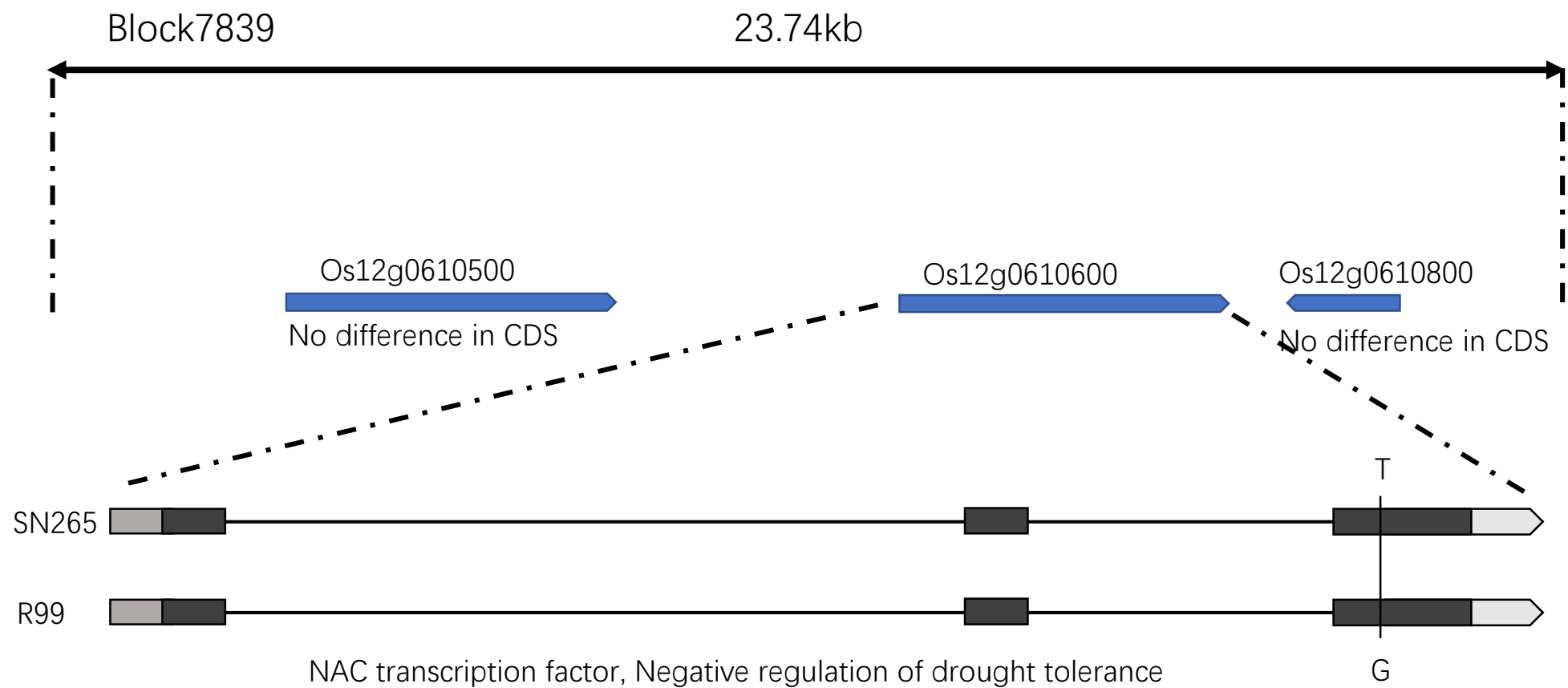

Supplement: Supplementary file 10 — Figure S5. Candidate gene prediction of grain shape regulate locus on chromosome 12. (PDF 23 kb) [file 12915_2018_572_MOESM10_ESM.pdf]

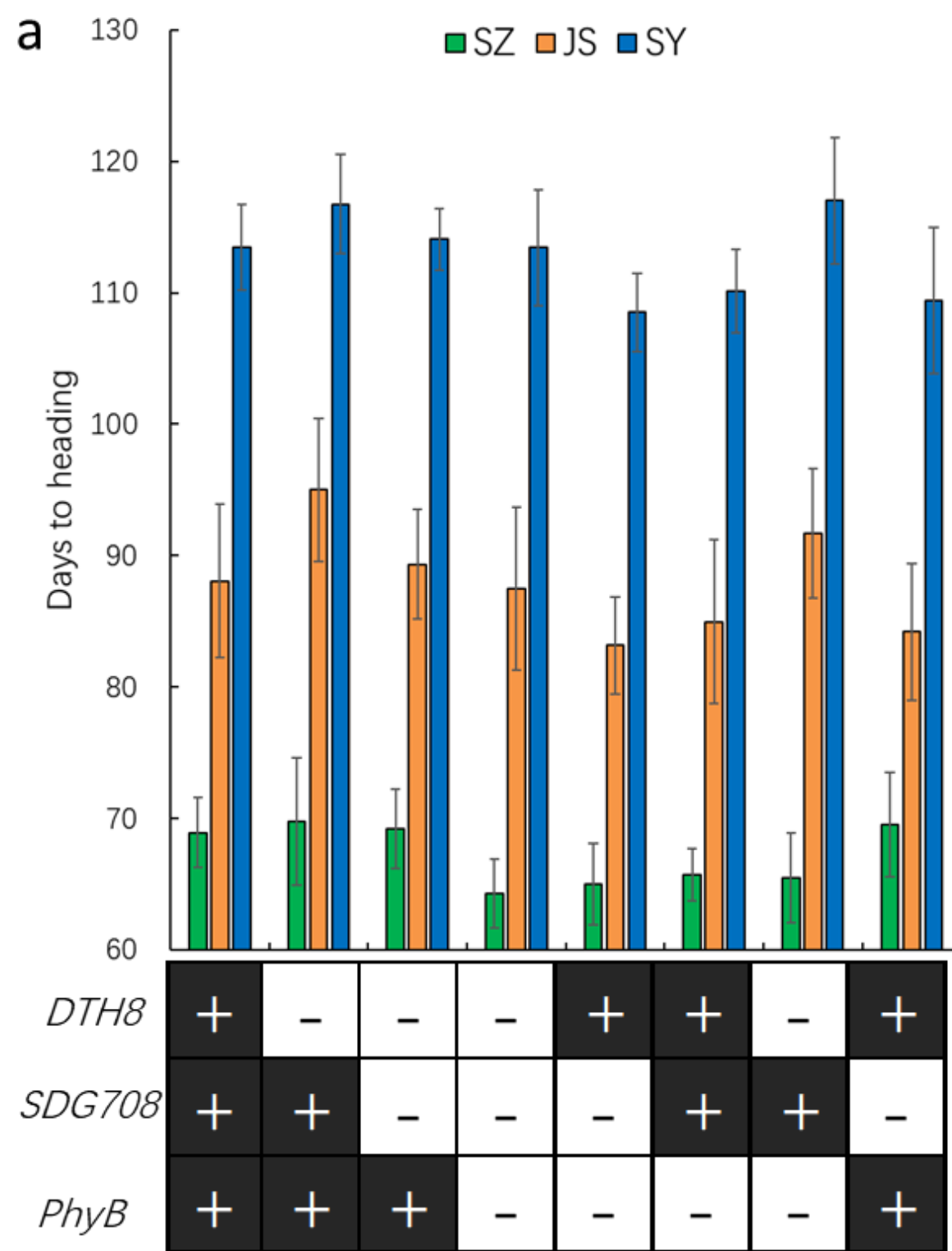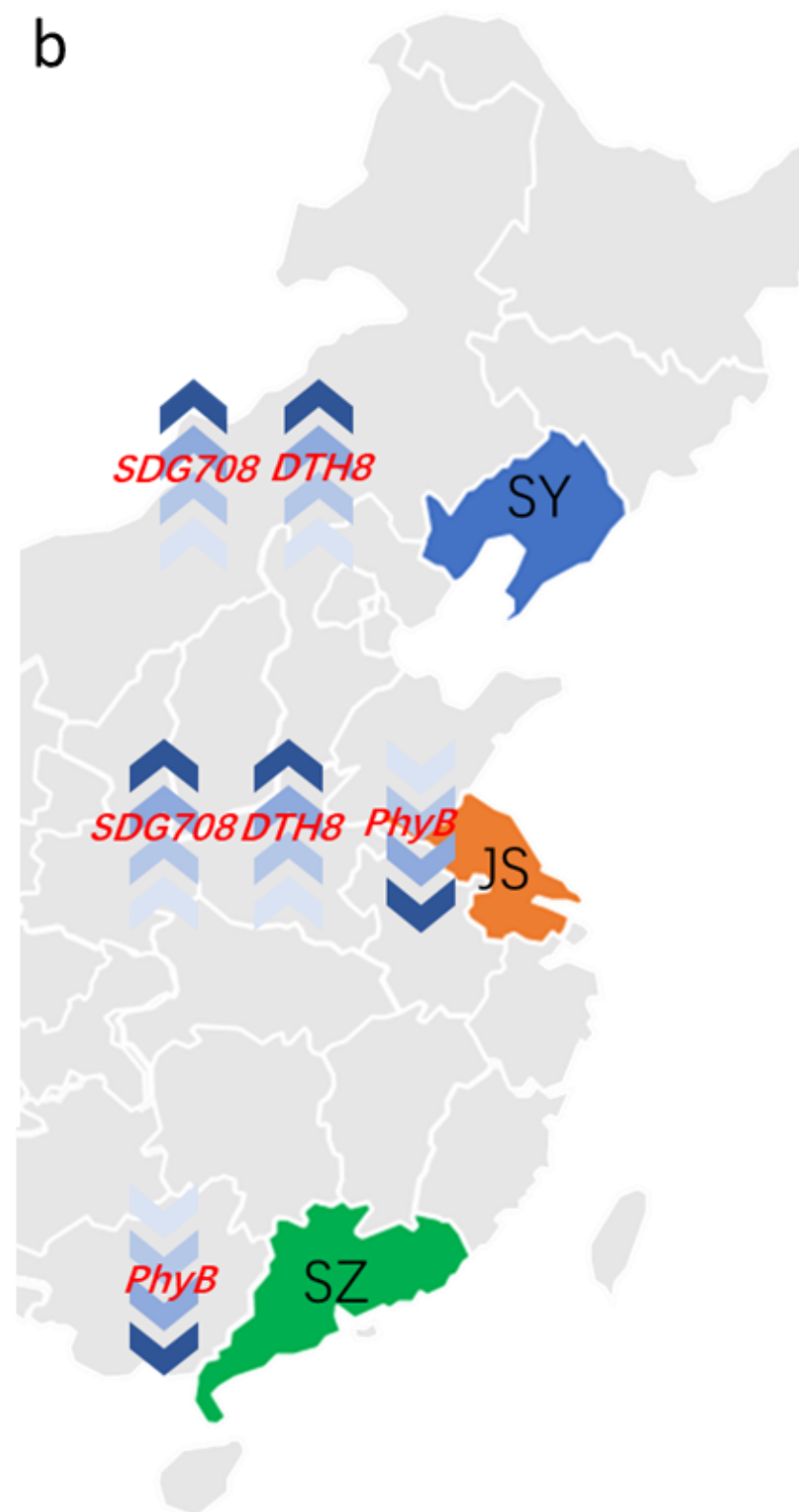

Supplement: Supplementary file 11 — Figure S6. The effects of DTH8, SDG708, and PHYB in different areas: a, the heading date of the different combination of DTH8, SDG708, and PHYB; b, the major effect heading gene in three areas. The data are the mean ± s.d. (n = 20 plants), “+” and “−” indicate the R99-type and S265-type alleles, respectively. (PDF 185 kb) [file 12915_2018_572_MOESM11_ESM.pdf]

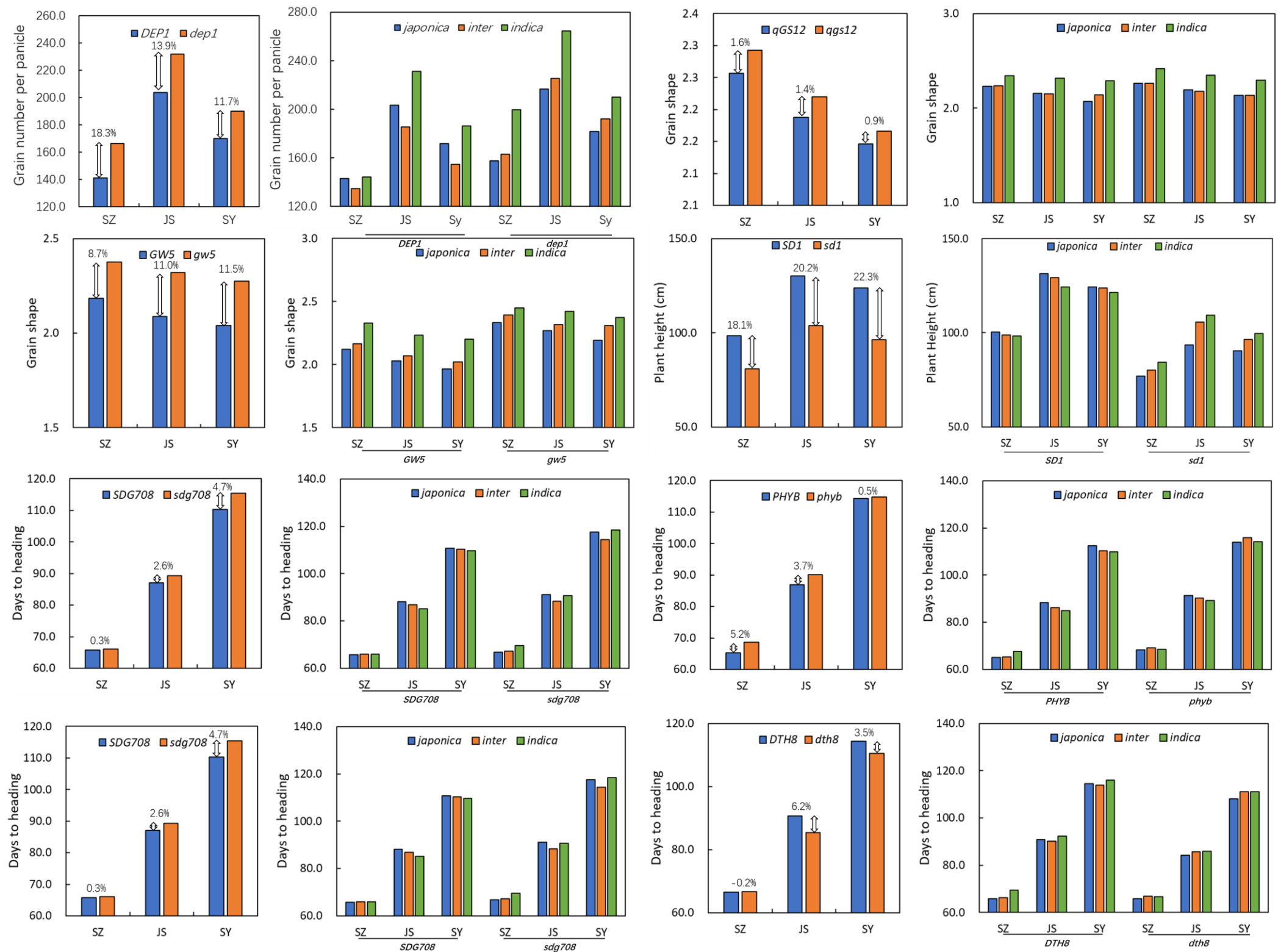

Supplement: Supplementary file 12 — Figure S7. The influence of ecological conditions and genetic background on gene function. The uppercase and lowercase lettered gene name indicates R99-type and SN265-type alleles, respectively. (PDF 378 kb) [file 12915_2018_572_MOESM12_ESM.pdf]

a

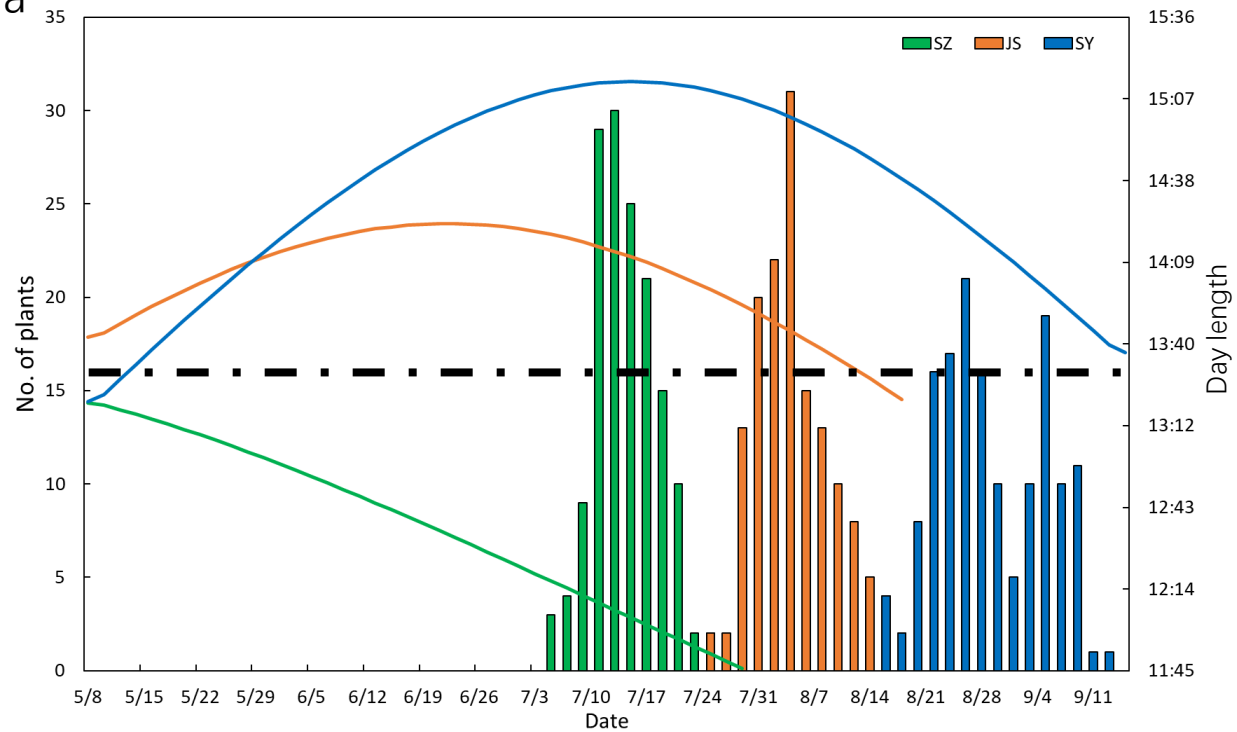

b

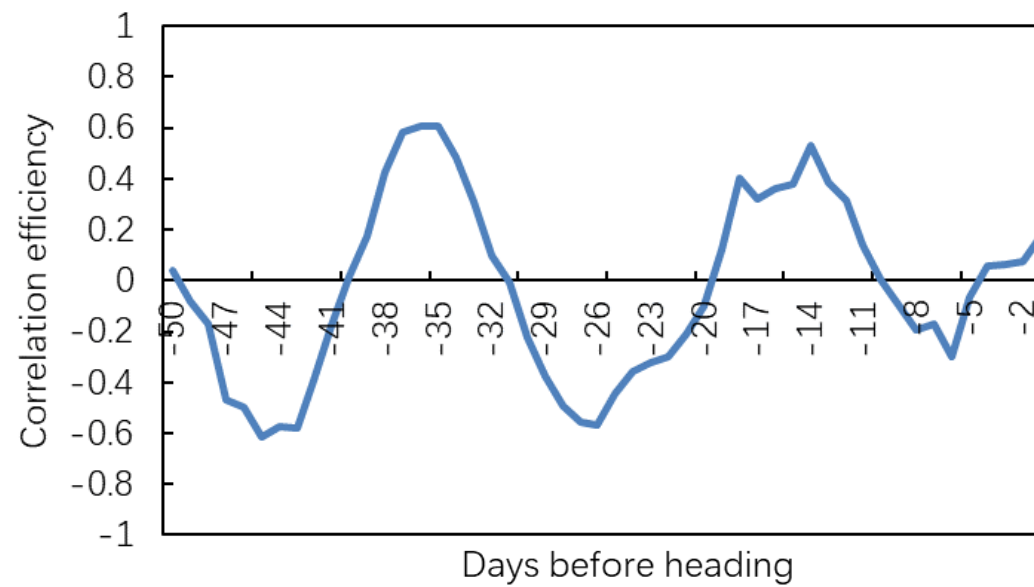

Supplement: Supplementary file 14 — Figure S8. The heading time of RILs in the three areas: a, the heading data of RILs and the day length in three areas; b, the correlation coefficient between air temperature and heading time. (PDF 109 kb) [file 12915_2018_572_MOESM14_ESM.pdf]
